# Retraction: Cadaveric Study of Anatomic Far Distal Musculocutaneous and Median Nerve Communication

**DOI:** 10.7759/cureus.r7

**Published:** 2016-09-09

**Authors:** Namath S Hussain

**Affiliations:** 1 Neurological Surgery, Loma Linda University Medical Center, Loma Linda, USA

A retraction was requested by the author, Namath S. Hussain, due to his assertion that "upon further review, the literature review was not compete and the results section underdeveloped." Cureus editorial staff has reviewed the article and communicated with both Dr. Hussain and members of Penn State University's Department of Neurosurgery, at which Dr. Hussain was employed at the time of this case. After careful consideration, Cureus agrees with Dr.Hussain's assessment and is thus retracting the article.

